# A Novel Single-Color FRET Sensor for Rho-Kinase Reveals Calcium-Dependent Activation of RhoA and ROCK

**DOI:** 10.3390/s24216869

**Published:** 2024-10-26

**Authors:** Allison E. Mancini, Megan A. Rizzo

**Affiliations:** Department of Pharmacology, Physiology, and Drug Development, University of Maryland School of Medicine, Baltimore, MD 21201, USA; allison.mancini@som.umaryland.edu

**Keywords:** RhoA, ROCK, FRET, biosensor, calcium signaling, fibroblast

## Abstract

Ras homolog family member A (RhoA) acts as a signaling hub in many cellular processes, including cytoskeletal dynamics, division, migration, and adhesion. RhoA activity is tightly spatiotemporally controlled, but whether downstream effectors share these activation dynamics is unknown. We developed a novel single-color FRET biosensor to measure Rho-associated kinase (ROCK) activity with high spatiotemporal resolution in live cells. We report the validation of the Rho-Kinase Activity Reporter (RhoKAR) biosensor. RhoKAR activation was specific to ROCK activity and was insensitive to PKA activity. We then assessed the mechanisms of ROCK activation in mouse fibroblasts. Increasing intracellular calcium with ionomycin increased RhoKAR activity and depleting intracellular calcium with EGTA decreased RhoKAR activity. We also investigated the signaling intermediates in this process. Blocking calmodulin or CaMKII prevented calcium-dependent activation of ROCK. These results indicate that ROCK activity is increased by calcium in fibroblasts and that this activation occurs downstream of CaM/CaMKII.

## 1. Introduction

Ras homolog family member A (RhoA) is a small GTPase that acts as a molecular switch and signaling hub, controlling integral processes, including migration, cell cycle progression, and cell division [1,2,3,4,5]. Like other small GTPases, RhoA cycles between an active, GTP-bound “on” state, localized to the membrane, and an inactive, GDP-bound “off” state, localized to the cytosol [6,7,8,9,10]. This cycling is controlled mainly by activating RhoGEFs that promote the exchange of GDP for GTP and inactivating RhoGAPs that enhance GTP hydrolysis [6,9,11,12,13,14,15,16,17]. Additionally, RhoGDIs bind and sequester inactive RhoA in the cytoplasm and extract RhoA from the membrane following GTP hydrolysis [6,18,19,20,21,22]. Together, these proteins respond to diverse signaling events to finely tune RhoA activity.

RhoA’s activity is mediated by several downstream effectors, with its major effector being Rho-associated kinase (ROCK). ROCK is a serine-threonine protein kinase that can phosphorylate its targets when bound to active RhoA. ROCK’s targets include adducin, ERM family members, LIM kinase, myosin light chain phosphatase, the Na^+^/H^+^ exchanger, and vimentin, among others [23,24,25]. ROCK has emerged in recent years as a potential therapeutic target in diseases including metastatic cancer, stroke, Alzheimer’s disease, schizophrenia, and spinal cord injury [26,27,28,29,30,31]. ROCK has also been implicated in disease processes such as drug resistance in leukemia, ovarian and breast cancer migration, and viral replication [32,33,34,35,36]. Clinically, Rho-kinase inhibitors manage ophthalmologic conditions, including glaucoma, ocular hypertension, and diabetic retinopathy; novel ROCK inhibitors for these purposes are frequently reported [37,38,39,40,41,42,43,44,45,46,47,48,49,50,51,52]. ROCK inhibitors have also been successfully used to manage vascular conditions such as erectile dysfunction and pulmonary hypertension, as well as to improve chronic graft-versus-host disease [53,54,55,56,57,58,59].

Förster resonance energy transfer (FRET) biosensors sensitively quantify protein activity in living cells, revealing the spatial and temporal dynamics of key molecular signals [60,61,62,63,64,65,66]. Typically, FRET sensors incorporate donor and acceptor fluorophores of different colors, notably cyan and yellow fluorescent proteins [61,62,63,67]. Changes in sensor activity alter the FRET efficiency, which is typically quantified by using the acceptor-to-donor fluorescence ratio. The direct relationship between FRET efficiency and sensor activity allows calibration and quantitative analyses [64,68,69,70]. Even so, a two-color, heterotransfer approach to FRET has several drawbacks, most notably the large portion of the visible spectrum taken up by using two differently colored fluorophores. These reporters are challenging to combine with other optical tools, such as channelrhodopsin, additional sensors, and organic fluorescent indicator dyes.

To improve quantification and multiplexing, we developed single-color FRET biosensors that measure a change in the polarization state rather than the color of the emitted light. These FLuorescence Anisotropy REporters (FLAREs) take advantage of the depolarizing effect of FRET [60] on the highly polarized fluorescence of large autofluorescent proteins [71,72]. Typically, these sensors include two fluorescent proteins of the same color added to a biosensing sequence that changes conformation upon triggering. FRET imaging reveals the difference between sensor states but is readout by measuring changes in emitted light polarization rather than a change in the color of emitted light, as happens with two-color FRET sensors. This change in polarization, quantified as fluorescence anisotropy, can be measured more sensitively than fluorescence intensity, leading to improved signal-to-noise ratios [71,73]. These single-color homotransfer reporters are especially well-suited for biosensor multiplexing, making FLAREs particularly useful for applications where the activity of two or more proteins need to be quantitated precisely in the same cell at the same time [72,73,74]. Restriction to a single color creates space in the visible spectrum for additional optical tools, including fluorescent indicator dyes, light-activated optogenetic reagents like channelrhodopsin, or even additional FLARE biosensors.

Because of the increasing interest in ROCK’s role in driving clinical pathologies and basic cell physiology, we developed a sensor that reports ROCK activity in living cells. Although two-color FRET-based biosensors exist for several members in the RhoA signaling cascade, including RhoA, RhoGEFs, RhoGAPs, RhoGDI, and ROCK, the two-color approach limits possibilities for multiplexing sensors to study the activity of multiple proteins in real time [75,76,77,78,79,80]. Furthermore, a previously reported two-color ROCK FRET sensor showed cross-reactivity to PKA, hampering its utility in studying ROCK specifically [80]. To investigate the role of ROCK in live cells in a spatiotemporally resolved manner using a polarization-based FRET approach, we have developed a single-color ROCK FLARE-type biosensor, the Rho-Kinase Activity Reporter (RhoKAR). The sensor gives a readout specific to ROCK activity and is insensitive to the activity of PKA. This sensor has also revealed calcium-dependent activation of ROCK, underscoring the utility of this tool in characterizing signaling behavior in live cells.

## 2. Materials and Methods

### 2.1. Plasmid and Construct Cloning

The RhoKAR sensor was generated using standard molecular cloning techniques. Briefly, an insert containing an FHA domain and the ROCK substrate from adducin was ordered as an oligo inserted in the pUC57-Kan backbone with XhoI and AgeI cut sites flanking the insert (GenScript, Piscataway, NJ, USA). The plasmid was transformed into JM109 competent *E. coli* cells (Promega, Madison, WI, USA) via standard heat shock technique. These cells were then grown in media containing kanamycin to select for cells carrying the plasmid. The plasmid was extracted using standard DNA isolation Miniprep and Midiprep kits (Qiagen, Germantown, MD, USA). Using XhoI and AgeI restriction enzymes on the isolated plasmid, the insert of interest was excised via enzymatic digest, gel purified, and then ligated into the tandem mVenus:mVenus FRET backbone previously developed by our lab from the pEGFP-C1 backbone (Clontech, San Jose, CA, USA) [81]. The presence of the insert in the correct location and orientation within the mVenus:mVenus tandem FRET backbone was confirmed with a test digest and visualization of an insert of the appropriate size via gel electrophoresis, as well as via Sanger sequencing.

The DNA sequence for the sensor is

ATGGTGAGCAAGGGCGAGGAGCTGTTCACCGGGGTGGTGCCCATCCTGGTCGAGCTGGACGGCGACGTAAACGGCCACAAGTTCAGCGTGTCCGGCGAGGGCGAGGGCGATGCCACCTACGGCAAGCTGACCCTGAAGCTGATCTGCACCACCGGCAAGCTGCCCGTGCCCTGGCCCACCCTCGTGACCACCCTGGGCTACGGCCTGCAGTGCTTCGCCCGCTACCCCGACCACATGAAGCAGCACGACTTCTTCAAGTCCGCCATGCCCGAAGGCTACGTCCAGGAGCGCACCATCTTCTTCAAGGACGACGGCAACTACAAGACCCGCGCCGAGGTGAAGTTCGAGGGCGACACCCTGGTGAACCGCATCGAGCTGAAGGGCATCGACTTCAAGGAGGACGGCAACATCCTGGGGCACAAGCTGGAGTACAACTACAACAGCCACAACGTCTATATCACCGCCGACAAGCAGAAGAACGGCATCAAGGCCAACTTCAAGATCCGCCACAACATCGAGGACGGCGGCGTGCAGCTCGCCGACCACTACCAGCAGAACACCCCCATCGGCGACGGCCCCGTGCTGCTGCCCGACAACCACTACCTGAGCTACCAGTCCAAACTGAGCAAAGACCCCAACGAGAAGCGCGATCACATGGTCCTGCTGGAGTTCGTGACCGCCGCCGGGATCACTCTCGGCATGGACGAGCTGTACAAGTCCGGACTCAGATCTCGAGATAAGTTTTCTCAAGAACAGATCGGCGAAAACATTGTGTGCAGGGTCATTTGTACCACGGGTCAAATTCCCATCCGAGATTTGTCAGCTGATATTTCACAAGTGCTTAAGGAAAAACGATCCATAAAGAAAGTTTGGACATTTGGTAGAAACCCAGCCTGTGACTATCATTTAGGAAACATTTCAAGACTGTCAAATAAGCATTTCCAAATACTACTAGGAGAAGACGGTAACCTTTTATTGAATGACATTTCCACTAATGGGACCTGGTTAAATGGGCAAAAAGTCGAGAAGAACAGCAATCAGTTACTGTCTCAAGGTGATGAAATAACCGTTGGTGTAGGCGTGGAATCAGATATTTTATCTCTGGTCATTTTCATAAACGACAAATTTAAGCAGTGCCTGGAGCAGAACAAAGTTGATCGCTCTGCAGGTAAGCCAGGCAGCGGCGAGGGCAGCACCAAGCAGCAGCGTGAAAAGACCCGCTGGCTGGGCGGCACCGGCGGCTCACCGGTCGCCACCATGGTGAGCAAGGGCGAGGAGCTGTTCACCGGGGTGGTGCCCATCCTGGTCGAGCTGGACGGCGACGTAAACGGCCACAAGTTCAGCGTGTCCGGCGAGGGCGAGGGCGATGCCACCTACGGCAAGCTGACCCTGAAGCTGATCTGCACCACCGGCAAGCTGCCCGTGCCCTGGCCCACCCTCGTGACCACCCTGGGCTACGGCCTGCAGTGCTTCGCCCGCTACCCCGACCACATGAAGCAGCACGACTTCTTCAAGTCCGCCATGCCCGAAGGCTACGTCCAGGAGCGCACCATCTTCTTCAAGGACGACGGCAACTACAAGACCCGCGCCGAGGTGAAGTTCGAGGGCGACACCCTGGTGAACCGCATCGAGCTGAAGGGCATCGACTTCAAGGAGGACGGCAACATCCTGGGGCACAAGCTGGAGTACAACTACAACAGCCACAACGTCTATATCACCGCCGACAAGCAGAAGAACGGCATCAAGGCCAACTTCAAGATCCGCCACAACATCGAGGACGGCGGCGTGCAGCTCGCCGACCACTACCAGCAGAACACCCCCATCGGCGACGGCCCCGTGCTGCTGCCCGACAACCACTACCTGAGCTACCAGTCCAAACTGAGCAAAGACCCCAACGAGAAGCGCGATCACATGGTCCTGCTGGAGTTCGTGACCGCCGCCGGGATCACTCTCGGCATGGACGAGCTGTACAAG.

The protein sequence for the sensor is

MVSKGEELFTGVVPILVELDGDVNGHKFSVSGEGEGDATYGKLTLKLICTTGKLPVPWPTLVTTLGYGLQCFARYPDHMKQHDFFKSAMPEGYVQERTIFFKDDGNYKTRAEVKFEGDTLVNRIELKGIDFKEDGNILGHKLEYNYNSHNVYITADKQKNGIKANFKIRHNIEDGGVQLADHYQQNTPIGDGPVLLPDNHYLSYQSKLSKDPNEKRDHMVLLEFVTAAGITLGMDELYK.

### 2.2. Cell Culture and Transfection

NIH-3T3 cells were maintained in Dulbecco’s Modification of Eagle’s Medium (DMEM) supplemented with 10% fetal bovine serum (Gibco, Waltham, MA, USA) and 1% penicillin/streptomycin (HyClone, Logan, UT, USA) and incubated at 37 °C with 5% CO_2_. Culture cell density was maintained according to ATCC recommendations. Cells were plated on 35-mm glass bottom dishes 24 h before transfection. Cells were transfected using 1 µg of DNA per plate using LipoD293 (SignaGen, Frederick, MD, USA) 48 h before imaging. Approximately 48 h after transfection, to prepare cells for imaging, growth media was removed from confluent cells immediately before imaging. Cells were then washed twice with 37 °C Hank’s Balanced Salt Solution (HBSS) (Gibco, Waltham, MA, USA) supplemented with 0.1% bovine serum albumin (BSA) (Sigma, St. Louis, MO, USA). After washes, cells were maintained in 2 mL of 37 °C HBSS with 0.1% BSA for imaging experiments. Where indicated, serum starvation was achieved by replacing the complete media with serum-free DMEM overnight before imaging. Drug treatments during imaging included lysophosphatidic acid (Abcam, Cambridge, UK), Y27632 (Sigma-Aldrich, St. Louis, MO, USA), Forskolin (Sigma-Aldrich), H89 (Cell Signaling Technology, Danvers, MA, USA), ionomycin calcium salt from *Streptomyces conglobatus* (Sigma-Aldrich, St. Louis, MO, USA), EGTA (Research Projects International, Mount Prospect, IL, USA), W-7 (MedChemExpress, Monmouth Junction, NJ, USA), and KN-93 (Sigma-Aldrich, St. Louis, MO, USA). Drug treatments were applied by manually pipetting the drug into the imaging media at the concentration indicated at T = 0 min. Equivalent volume vehicle control treatments were used as negative controls for all drug treatment experiments. For any drug pretreatment, the drug was added at the indicated concentration into the imaging media immediately after washes.

### 2.3. Fluorescence Polarization Microscopy

Fluorescence polarization methods are described in detail in previous publications [72,74]. Briefly, two Zeiss AxioObserver Microscopes (Carl Zeiss MicroImaging, Oberkochen, Germany) equipped for widefield imaging were used to gather fluorescence anisotropy images. The Zeiss AxioObserver Z1 microscope was controlled using Zeiss AxioVision 4.7.2 software, and the Zeiss AxioObserver 7 was controlled using Zeiss Zen Blue 2.6 software. Images were collected with a 20X 0.75 NA objective lens on the Zeiss AxioObserver Z1 microscope. This setup included a 505 nm LED illumination source, followed by a wire grid polarizer (Meadowlark Optics, Frederick, CO, USA) in the excitation pathway before a filter turret containing a YFP filter cube (Zeiss, Oberkochen, Germany). Light from the sample passed through a DualView beam splitter (Optical Insights, Exton, PA, USA) equipped with filters for the parallel and perpendicular states of light. Both polarization states were collected simultaneously as a single image by an Orca-R2 water-cooled camera (Hamamatsu, Shizuoka, Japan). The Zeiss AxioObserver 7 had a nearly identical setup but used a Gemini W-View (Hamamatsu) as a beam splitter before light collection with an AxioCam 506 (Zeiss) camera. Where indicated, a 40X 1.3 NA oil immersion objective lens was used for imaging on the second microscope.

The microscopes used were fitted with XL-1 Dark S1 light-tight incubators (Pecon) custom-made for Zeiss AxioObserver microscopes. Cells were maintained in normal atmospheric gas. Temperatures inside the microscope incubators were maintained at 37 °C by a heated stage insert, a Heating Unit XLS (Zeiss), and a TempModule S (Zeiss). For all time course imaging experiments, images were taken every 30 s for the duration of the experiment. Cells were imaged for several minutes before the addition of any drugs to establish baseline anisotropy values.

### 2.4. Heterotransfer FRET Microscopy

We also used a two-color FRET biosensor for RhoA activity, the RhoA-mCer3 biosensor that we have previously reported [75]. The RhoA-mCer3 sequence and sensor schematic are presented in Supplemental Figure S1. For CFP/YFP FRET imaging, a 455 nm LED was used for sample illumination. The same microscope setup described above for polarization microscopy was used with a CFP/YFP FRET filter cube (Zeiss) as well as a CFP/YFP filter set in the DualView immediately before the camera.

### 2.5. Image Analysis

Widefield images of different polarization states were split into separate images, then recombined into a single two-channel image and aligned using the StackReg plugin package in FIJI [82]. Background-corrected fluorescence intensity measurements were taken at each time point in individual cells using hand-drawn ROIs. Fluorescence anisotropies were calculated as described previously. Briefly, fluorescence anisotropy (R) is calculated as a ratio between the intensities of the two polarization states of light, parallel (P) and perpendicular (S), over the total fluorescence intensity:

(1) $$R=\frac{P-gS}{P+2gS}$$

where (g) corrects for differences in camera sensitivity to each polarization state of light. After collecting fluorescence intensity measurements in FIJI, fluorescence anisotropies were calculated in Microsoft Excel (Microsoft 365 Office for Mac) Anisotropy values were smoothed in GraphPad Prism 7 before the change in fluorescence anisotropy over baseline was calculated for each individual cell in Microsoft Excel. Statistical analysis and figure generation were done using GraphPad Prism. Because a decrease in anisotropy (R) corresponds to an increase in ROCK activity for the RhoKAR sensor, fluorescence anisotropies are presented as −R in this paper.

For CFP/YFP FRET images, pre-processing was performed as described above for polarization-based FRET images. After background correction, FRET was calculated as a bleedthrough corrected ratio between CFP (C) and YFP (Y) intensities using the following equation:

$$FRET\;Ratio=\frac{Y-bC}{C}$$

where (b) corrects for CFP fluorescence that bleeds through into the YFP channel due to overlap between the CFP and YFP emission spectra.

For LUT image creation, background-corrected fluorescence anisotropies were calculated in each pixel within FIJI using the above equation, multiplied by −1 to get −R, backgrounds were removed, images were smoothed, and the MPL-Inferno LUT was applied to create FRET map images. LUT images for the RhoA-mCer3 CFP/YFP FRET sensor were generated as described above using the calculated CFP/YFP FRET ratio rather than −R. Figures were compiled in BioRender and Microsoft PowerPoint (Microsoft Office 365 for Mac).

## 3. Results

### 3.1. Development and Validation of Single-Color RhoKAR FRET Biosensor

**Figure 1:**
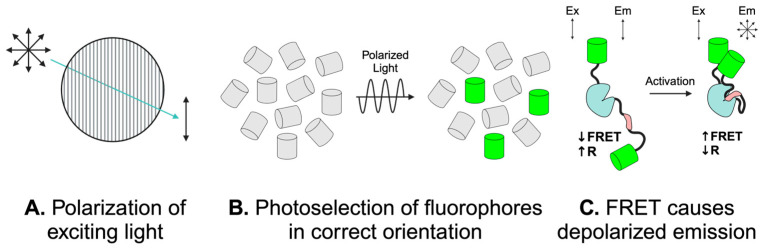
Anisotropy-based FRET strategy. (**A**) Light, represented by the blue arrow, passes through a polarized filter, so all exciting light is in the same polarization state. Polarization states of light are represented by the intersecting or individual double-sided arrows. (**B**) The polarized light excites fluorophores, represented as cylinders, in the correct geometric orientation. Fluorophores in the correct geometric orientation that are photoselected and, therefore, excited by the uniformly polarized light are represented in green. (**C**) In the absence of FRET, emission (Em) from photoselected fluorophores will be in the same polarization state as exciting light (Ex). Upon conformational change of the biosensor associated with activation, FRET to a nearby acceptor fluorophore causes depolarized emission, as measured by a lowered anisotropy (R). Adapted from Snell et al. [73]. Created in BioRender. Mancini, A. (2024) BioRender.com/l35z225.

FLAREs use two fluorophores of a single color to provide homotransfer-FRET readouts based on the change in the polarization state of emitted light. Fluorescence emission from fluorophores is highly polarized or anisotropic [71]. When light in a uniform polarization state excites a fluorophore, only fluorophores in the correct geometric orientation in space are excited via a process known as photoselection (Figure 1A,B) [73]. Emission from photoselected fluorophores will be in the same polarization state as the exciting wavelength in the absence of energy transfer to a nearby acceptor fluorophore. However, emission is depolarized if energy transfer occurs to an acceptor fluorophore within a molecular distance (Figure 1C). This technique enables us to design single-color FRET biosensors that provide real-time protein activity readouts in live cells. Fluorescence anisotropies can also be measured more sensitively than fluorescence intensity, allowing for precise quantification of small shifts in FRET ratios [71]. FLAREs do not require bleedthrough correction and can be more easily multiplexed than two-color FRET sensors [73]. FLAREs can be used on standard widefield microscopes using a linear polarizer in the light path before excitation of the sample and a polarization-splitting optical filter in front of the camera. This setup allows for the collection of light in both polarization states simultaneously to get fluorescence anisotropy readouts of biosensor activity [72,75].

**Figure 2:**
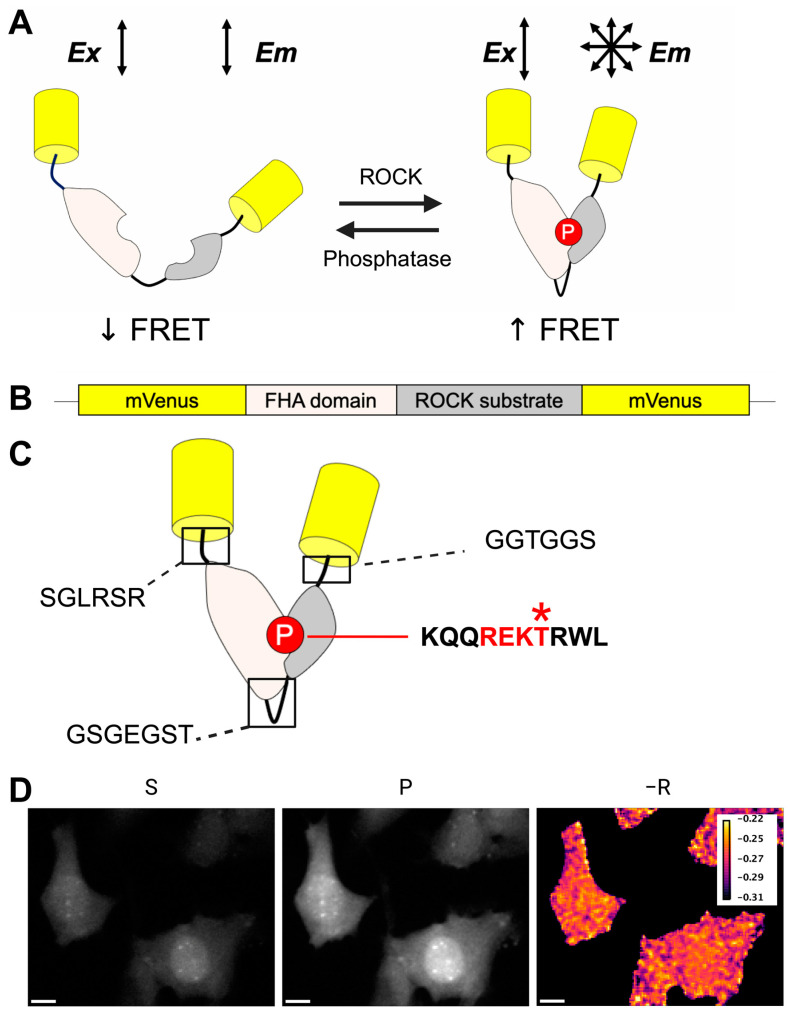
RhoKAR sensor schematic. (**A**,**B**) The RhoKAR sensor consists of two mVenus fluorophores flanking an FHA domain and the ROCK substrate from adducin. When ROCK activity is low, the ROCK substrate is unphosphorylated, so the FHA domain is unable to interact with it, causing low FRET. When ROCK is activated, it phosphorylates the substrate, leading to a conformational change and an increase in FRET. (**C**) Schematic showing amino acid sequences for linker regions and ROCK phosphorylation motif (bolded with red line; * indicates phosphorylated site. (**D**) Representative images of NIH-3T3 cells expressing the RhoKAR sensor in the Perpendicular (S) and Parallel (P) polarization state channels and pseudo-colored FRET map (−R), where brighter color indicates higher ROCK activity. Scale bar = 10 µm. Created in BioRender. Mancini, A. (2024) BioRender.com/b76g649.

We developed a single-color FRET sensor, the RhoKAR sensor, to measure ROCK activity directly. RhoKAR consists of two mVenus molecules flanking an FHA domain and the phosphorylation consensus sequence from adducin, a target of ROCK (Figure 2A,B). The adducin substrate sequence contains the −6 to +3 amino acids surrounding Threonine 445 (Figure 2C). This sequence does not contain any other amino acids that can be phosphorylated. Adducin Threonine 445 has been shown to be phosphorylated only by ROCK [83,84,85]. Activated ROCK phosphorylates the sensor’s substrate, resulting in binding to the adjacent FHA domain and a conformational change that increases FRET. De-phosphorylation of the substrate by intracellular phosphatases, such as MLCP, converts the sensor back into an inactive state [86,87,88,89,90]. Images of the sensor were collected in the perpendicular (S) and parallel (P) polarization states (Figure 2D). We then calculated fluorescence anisotropies (−R) using the difference in intensities in the two channels. We visualized RhoKAR activity throughout the cell with a pseudo-colored FRET map image generated in FIJI (Figure 2D).

### 3.2. RhoKAR Provides a Readout of ROCK Stimulation and Inhibition

**Figure 3:**
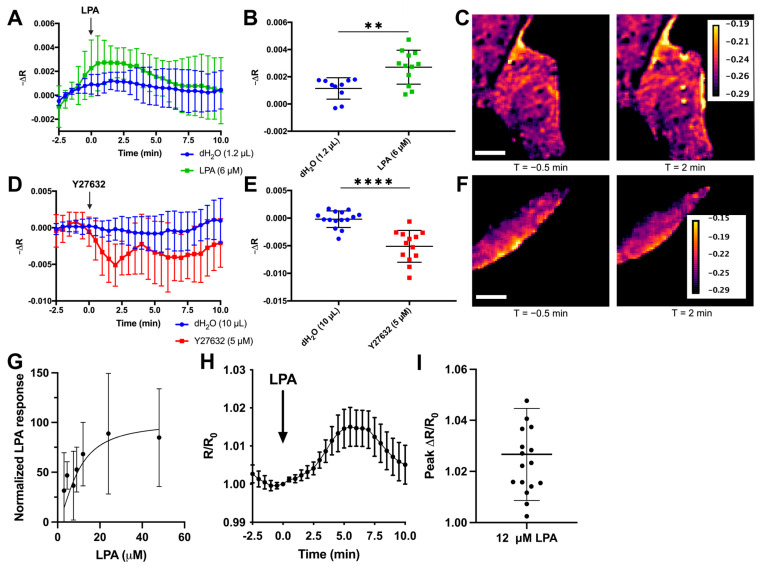
RhoKAR sensor gives a readout of ROCK stimulation and inhibition. (**A**,**B**) Time-course change in fluorescence anisotropies in individual NIH-3T3 cells expressing the RhoKAR sensor when stimulated with 6 µM LPA or 1.2 µL dH_2_O vehicle control (*t*-test, ** *p* < 0.01, n = 10 for dH_2_O, n = 12 for LPA). (**C**) Representative FRET map image of a cell immediately before and 2 min after LPA treatment. Scale bar = 10 µm. (**D**,**E**) Time-course change in fluorescence anisotropies in individual cells expressing the RhoKAR sensor when stimulated with Y27632 or dH_2_O vehicle control (*t*-test, **** *p* < 0.0001, n = 15 for dH_2_O, n = 13 for Y27632). (**F**) Representative FRET map of cells immediately before and 2 min after Y27632 treatment. Scale bar = 10 µm. (**G**) Normalized dose-response of RhoKAR to increasing doses of LPA with curve fit (r^2^ = 0.02). (**H**) Increase in RhoKAR anisotropy compared to T = 0 following stimulation with 12 µM LPA. (**I**) Peak increase in anisotropy compared to T = 0 min after 12 µM LPA stimulation. Created in BioRender. Mancini, A. (2024) BioRender.com/r74d317.

To determine whether the RhoKAR sensor responds appropriately to changes in ROCK activity, we stimulated serum-starved cells expressing RhoKAR with 6 µM lysophosphatidic acid (LPA), a classical RhoA activator [91], during a short imaging time course (Figure 3A). We imaged cells for several minutes before drug addition to establish baseline anisotropies and added the indicated drug at T = 0 min. The average fluorescence anisotropy in each cell in the 3 min before drug addition was subtracted from the fluorescence anisotropy in that same cell at each time point to yield a change in fluorescence anisotropy over baseline (−∆R). LPA treatment significantly increased the change in fluorescence anisotropy over baseline in the RhoKAR sensor compared to equivalent volume dH_2_O vehicle control (Figure 3B). This corresponds to an increase in phosphorylation of the adducin substrate on RhoKAR by ROCK. Intensity maps with FRET map overlays show increased ROCK activity at 2 min compared to the time point immediately before LPA addition (Figure 3C). Next, we treated cells with 5 µM Y27632, a selective ATP-competitive inhibitor of ROCK (Figure 3D). Y27632 treatment significantly decreased RhoKAR activity compared to vehicle control (Figure 3E). FRET map images illustrate the decrease in RhoKAR activity seen with the Y27632 treatment (Figure 3F). To explore the dynamic range of the sensor, we also treated serum-starved cells expressing the RhoKAR sensor to increasing doses of LPA. The integrated −∆R response from 5 to 10 min following stimulation was normalized for agonist versus response and curve fit from 0–100, yielding a curve with an r^2^ of 0.02. These data show a dose-dependent response in RhoKAR activity to LPA stimulation (Figure 3G). To calculate the dynamic range of the sensor, the response to 12 µM LPA was calculated at each time point as −R from that time point divided by −R at T = 0 min, yielding R/R_0_ values (Figure 3H). Peak R/R_0_ values after 12 µM LPA stimulation showed an average change of 2.7%, with a maximum change of 7.8% observed (Figure 3I). Because the measured anisotropy shifts as expected when ROCK is stimulated or inhibited, these results show that the RhoKAR sensor gives a readout of ROCK activation and de-activation in live cells.

### 3.3. RhoKAR Gives a Readout Specific to ROCK Activity

**Figure 4:**
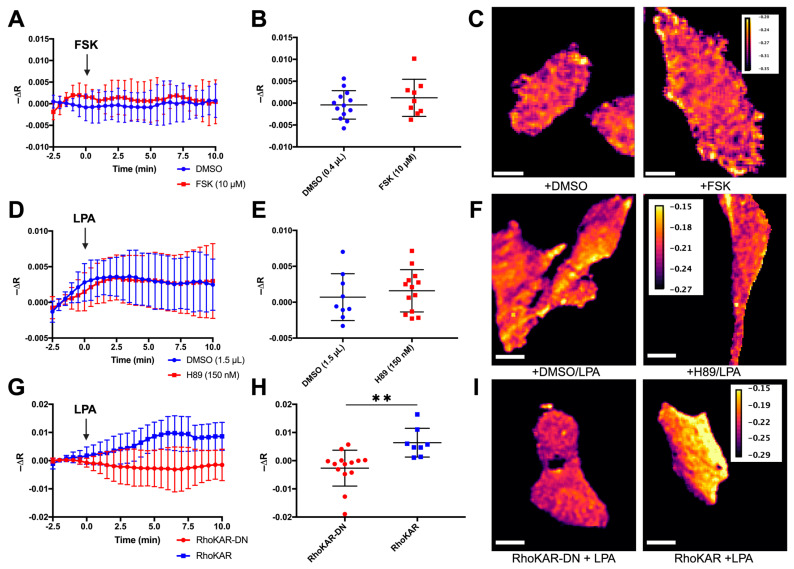
RhoKAR sensor readout is specific to ROCK activity. (**A**,**B**): Time-course change in fluorescence anisotropies in individual NIH-3T3 cells expressing the RhoKAR sensor when stimulated with Forskolin or DMSO vehicle control (*t*-test, ns; n = 13 for DMSO, n = 9 for FSK). (**C**) Representative FRET map images of cells 5 min after treatment with DMSO or FSK. Scale bar = 10 µm. (**D**,**E**) Time-course change in fluorescence anisotropies in individual serum-starved cells expressing the RhoKAR sensor when pretreated with 150 nM H89 or 1.5 µL DMSO vehicle control and stimulated with LPA (*t*-test, ns; n = 9 for DMSO, n = 13 for H89). (**F**) Representative FRET map images of cells pretreated with DMSO or H89 5 min after LPA stimulation. Scale bar = 10 µm. (**G**,**H**) Time-course change in fluorescence anisotropies in individual serum-starved cells expressing the RhoKAR wild-type sensor (blue) or the RhoKAR-DN phospho-dead mutant sensor after stimulation with 6 µM LPA (*t*-test, ** *p* < 0.01; n = 14 for RhoKAR-DN, n = 8 for RhoKAR). (**I**) Representative FRET map images of cells expressing the RhoKAR-DN or RhoKAR sensor 5 min after LPA stimulation. Scale bar = 10 µm. Created in BioRender. Mancini, A. (2024) BioRender.com/q90m740.

A previous ROCK sensor cross-reacted with PKA, reducing its utility in studying ROCK activity specifically [80]. To validate that RhoKAR readout is insensitive to PKA activity, 10 µM forskolin (FSK), a PKA activator was used to stimulate cells (Figure 4A). FSK stimulation did not significantly change RhoKAR activity compared to vehicle control (Figure 4B), indicating that RhoKAR is insensitive to PKA activity (Figure 4C). In serum-starved cells, we next inhibited PKA using 150 nM H89 and then stimulated cells with LPA during time-course imaging (Figure 4D). We did not observe significant differences in fluorescence anisotropies between the H89 and DMSO pretreated cells after LPA stimulation (Figure 4E,F), indicating that sensor activity is also insensitive to PKA inhibition.

Next, we generated a non-phosphorylatable mutant version of the sensor, the RhoKAR-DN sensor, with a threonine-to-alanine substitution at the phosphorylation site. Cells expressing the mutant or wild-type sensor were stimulated with 6 µM LPA after serum starvation (Figure 4G). While the wild-type RhoKAR sensor responded to LPA stimulation, the RhoKAR-DN sensor was insensitive to stimulation with 6 µM LPA. RhoKAR-DN showed a significantly lower change in time-course fluorescence anisotropies in individual cells after LPA stimulation compared to the wild-type sensor (Figure 4H,I). Together, these data show that RhoKAR specifically reports ROCK activity and that the shifts we observe in anisotropies correspond to conformational change following phosphorylation of ROCK substrate on the biosensor.

### 3.4. RhoKAR Visualizes Subcellularly Compartmentalized Activation of ROCK

**Figure 5:**
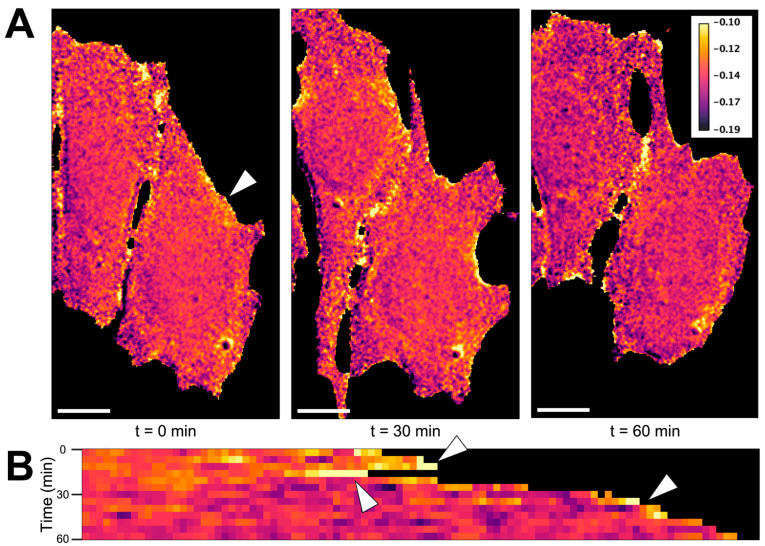
RhoKAR sensor allows for visualization of subcellular compartmentalized activation of ROCK. (**A**) NIH-3T3 cells expressing the RhoKAR sensor were imaged at 40X, with one image taken every 5 min for 1 h. Ratio images were generated and pseudo-colored to show areas of high RhoKAR activity. During the time course, one area of the cell membrane, indicated by an arrow, protruded and retracted. Scale bar = 10 µm. (**B**) A kymograph was generated for pixels near the edge of the cell near the arrow in the leftmost panel in A, showing RhoKAR activity through cell membrane protrusion and retraction during the course of imaging. Areas of high RhoKAR activity in the kymograph are demarked with arrows. Localized areas of high ROCK activity are observed during the process of cell protrusion and retraction. Created in BioRender. Mancini, A. (2024) BioRender.com/x61j261.

Spatial compartmentalization is an integral part of cellular processes, including GPCR engagement, endocytosis, and signaling for PKA, cAMP, and calcium [92,93,94,95,96]. Imaging techniques allowing for visualization of these dynamics has been utilized to study cyclic nucleotide signaling, small GTPase activity, as well as membrane and cytoskeletal dynamics [97,98,99,100]. RhoA activity is tightly regulated with subcellularly localized activation during cleavage furrow formation during cytokinesis, cell adhesion, endothelial barrier remodeling, and cell migration [15,101,102,103,104]. Whether ROCK follows the same activation patterns is unknown. To our knowledge, no biosensor exists that allows for quantitating ROCK activity, specifically with high spatiotemporal resolution without crosstalk from other kinases. To visualize the dynamics of ROCK activity at a subcellular level, we imaged fibroblasts expressing the RhoKAR sensor over a 1-hr time course at 40X magnification. LUT FRET map images were generated for each image in the time course. (Figure 5A). A line ROI was drawn at approximately the location of the arrow in leftmost panel in part A. Pixel intensities at each time point were stacked to create the kymograph. A movie showing the LUT images at each time point with an overlay of the ROI used to generate the kymograph is presented in Supplemental Video S1. Compartmentalized activation of ROCK was apparent, with areas of high ROCK activity observed during membrane protrusion and retraction over the imaging time course (Figure 5B). These data show that the RhoKAR sensor can visualize ROCK activity at the subcellular level.

### 3.5. Calcium Activates RhoA and ROCK in Fibroblasts

Calcium (Ca^2+^) is a ubiquitous second messenger in eukaryotic cells [105,106,107,108]. RhoA and ROCK’s role in sensitizing the contractile apparatus to calcium by inactivating MLCP has been well-characterized [109,110,111,112,113,114,115,116,117,118,119,120,121,122].

There have been other recent reports of calcium-dependent activation of RhoA in a variety of cell types [123,124,125,126,127,128,129,130,131]. Due to these findings, we were interested in exploring whether Ca^2+^ activates RhoA and ROCK in fibroblasts.

**Figure 6:**
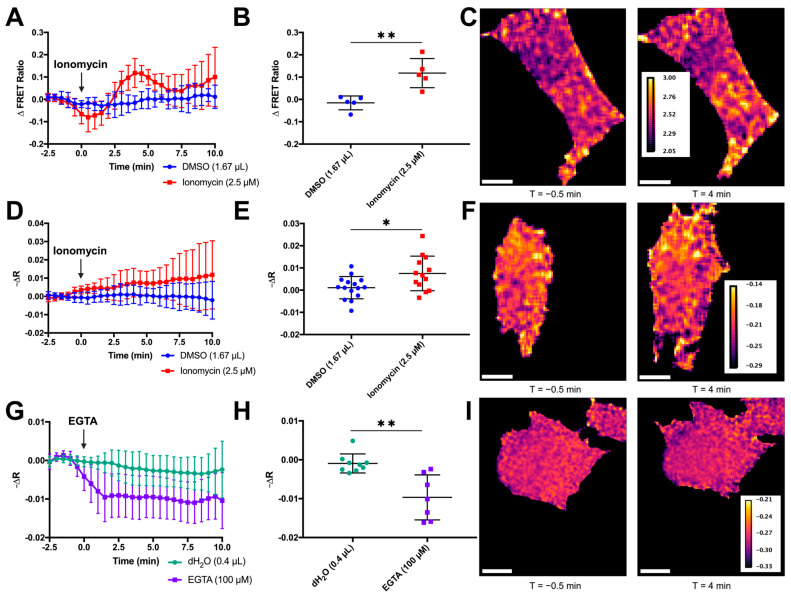
Calcium activates RhoA and ROCK in NIH-3T3 fibroblasts. (**A**,**B**) Time course change in FRET in single cells expressing the RhoA two-color FRET sensor shows a significant increase in RhoA activity after ionomycin stimulation compared to DMSO vehicle control (n = 5 per treatment group, *t*-test ** *p* < 0.01). (**C**) Representative FRET map images of cells 4 min after treatment with ionomycin. Scale bar = 10 µm. (**D**,**E**) Time course changes in fluorescence anisotropies were calculated for individual cells expressing the RhoKAR sensor after stimulation with ionomycin or DMSO vehicle control. (n = 15 for DMSO, n = 13 for ionomycin, *t*-test * *p* < 0.05). (**F**) Representative FRET map images of cells 4 min after treatment with ionomycin. Scale bar = 10 µm. (**G**,**H**) Time course change in fluorescence anisotropy in individual cells expressing the RhoKAR sensor after EGTA or dH_2_O vehicle control treatment (n = 9 for dH_2_O, n = 7 for EGTA, ** *p* < 0.01). (**I**) Representative FRET map images of cells 4 min after treatment with EGTA. Scale bar = 10 µm. Created in BioRender. Mancini, A. (2024) BioRender.com/p36e974.

We applied ionomycin calcium salt (IM), a calcium ionophore, to cells expressing our RhoA-mCer3 two-color FRET sensor [75]. This sensor consists of the Rho-binding domain (RBD) from Rhotekin and RhoA flanking an mCerulean3 donor fluorophore and a YFP acceptor fluorophore (Supplemental Figure S1). Activation of RhoA by exchange of GDP for GTP enables the RBD to bind RhoA-GTP, leading to a conformational change that causes an increase in FRET. This is measured as an increase in YFP emission compared to CFP emission. Ionomycin stimulation increased the activity of both the RhoA-mCer3 sensor (Figure 6A) and the RhoKAR sensor (Figure 6D). Ionomycin has been used extensively in the literature to increase intracellular calcium in live cells, including in fibroblasts [105,132,133]. Both the RhoA-mCer3 (Figure 6B,C) and RhoKAR (Figure 6E,F) sensors showed significant increases in FRET after 2.5 µM IM treatment compared to DMSO vehicle control, indicating that Ca^2+^ stimulation activates both RhoA and ROCK in fibroblasts. Interestingly, while the increase in RhoKAR activity was continuous, RhoA activation after ionomycin stimulation occurred in a biphasic manner (Figure 6A). We observed an initial peak of RhoA activity 4 min after ionomycin stimulation before a decrease in RhoA activity from around 6.5 to 7.5 min. This was followed by a second increase in RhoA activity beginning around 8 min. Notably, during the dip in RhoA activity between the two increases, RhoA activity does not return to baseline and is still greater than the RhoA activity observed in the DMSO vehicle control-treated cells. This residual RhoA activity above baseline could be driving the ROCK activity observed with the RhoKAR sensor during the decrease in RhoA activity. Biphasic RhoA activation has been observed in other systems, including in response to calcium influx [134,135].

Both positive and negative regulation by calcium is observed in hormone secretion and store-operated calcium channel conductivity, among other processes [136,137,138]. To see whether ROCK activity also shifted in response to decreasing Ca^2+^, we treated cells with 100 µM EGTA to reduce Ca^2+^ availability (Figure 6G). EGTA treatment significantly decreased RhoKAR activity compared to dH_2_O vehicle control (Figure 6H,I). This illustrates that reducing intracellular calcium decreases in ROCK activity. Together, these results indicate that RhoA and ROCK activity in NIH-3T3 fibroblasts is calcium-dependent.

### 3.6. Calcium Activation of ROCK in Fibroblasts Requires Calmodulin/CaMKII

**Figure 7:**
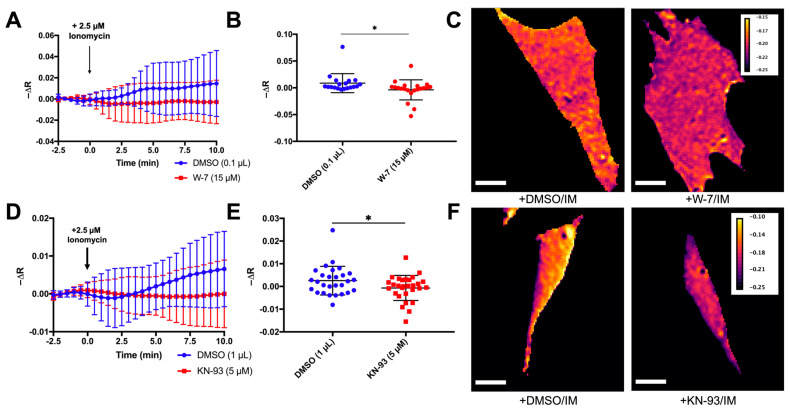
Calcium-dependent activation of ROCK requires Calmodulin and CaMKII. (**A**,**B**) Time course change in fluorescence anisotropies were calculated in individual cells expressing the RhoKAR sensor after pretreatment with DMSO or W-7 and stimulation with ionomycin at T = 0 min (n = 19 for DMSO/IM, n = 21 for W-7/IM, *t*-test * *p* < 0.05). (**C**) Representative FRET map images of cells pretreated with DMSO or W-7 4 min after stimulation with ionomycin. Scale bar = 10 µm. (**D**,**E**) Time course change in fluorescence anisotropies were calculated in individual cells expressing the RhoKAR sensor after pretreatment with DMSO or KN-93 and stimulation with ionomycin at T = 0 min (n = 28 for DMSO/IM, n = 29 for KN-93/IM, *t*-test * *p* < 0.05). (**F**) Representative FRET map images of cells pretreated with DMSO or KN-93 5.5 min after stimulation with ionomycin. Scale bar = 10 µm. Created in BioRender. Mancini, A. (2023) BioRender.com/s12v154.

Following our observation of calcium-dependent activation of RhoA and ROCK in mouse fibroblasts, we wanted to determine the underlying signaling mechanism. Calmodulin (CaM) is a ubiquitously expressed and highly conserved protein that undergoes a conformational change when calcium binds to any of its four binding sites [139,140,141]. Active CaM in complex with Ca^2+^ can relieve the autoinhibition of Calmodulin-dependent kinase II (CaMKII), a serine-threonine protein kinase and major effector of CaM [141,142]. CaM and CaMKII drive many physiological processes, including cytoskeletal rearrangement, apoptosis, and cell proliferation [140,143,144]. Another group has reported that ionomycin stimulation activates RhoA in rabbit aortic vascular smooth muscle and that this stimulation was sensitive to the inhibition of calmodulin but not CaMKII [145]. Others have observed that CaMKII inhibition blocks RhoA activation following glutaminergic calcium influx in dendritic spines [126]. To probe the role of CaM in Ca^2+^-dependent activation of ROCK in fibroblasts, we pretreated cells expressing the RhoKAR sensor with W-7, a CaM inhibitor, or DMSO vehicle control, then stimulated with 2.5 µM ionomycin (Figure 7A). W-7 pretreatment significantly decreased RhoKAR response to ionomycin stimulation compared to DMSO vehicle control pretreatment (Figure 7B,C), indicating that ionomycin-stimulated activation of ROCK requires calmodulin. Next, we chose to look downstream at CaMKII to determine whether calcium-dependent activation of ROCK in mouse fibroblasts requires this effector. KN93, a CaMKII inhibitor, was applied before stimulation with 2.5 µM ionomycin in cells expressing the RhoKAR sensor (Figure 7D). Compared to a DMSO vehicle control, KN93 pretreatment prevented ROCK activation by ionomycin (Figure 7E,F), indicating that CaMKII is involved in RhoA/ROCK activation downstream of ionomycin stimulation and CaM activity in mouse fibroblasts.

## 4. Discussion

### 4.1. The RhoKAR Sensor Enables Sensitive Quantification of ROCK Activity in Live Cells

Fluorescence and colorimetric-based assays have been developed for sensing protein localization and activity, biological metabolites, metal ions, and more [61,62,63,71,72,146,147,148,149,150,151,152,153]. FRET-based kinase activity reporter (KAR) type sensors have been developed for various kinases, including AKAR for PKA, CKAR for PKC, and EKAR for ERK [154,155,156]. These sensors have proven highly useful in studying cell signaling dynamics [157,158,159,160,161]. A previous FRET-based KAR-type sensor for ROCK used a two-color FRET approach. However, this sensor showed cross-reactivity to other kinases [80]. Other approaches available for monitoring ROCK are limited to cell-free methods such as ELISA kits or Western blot of cell lysates, recombinant enzyme assays using phosphosensitive fluorophores, and ATP radiolabeling assays [162,163,164,165]. While these cell-free approaches are useful, they don’t allow for quantification of ROCK activity in live cells, don’t offer spatial resolution of ROCK activity at the single-cell or subcellular level, and are not as well time-resolved as fluorescence-based approaches.

To monitor ROCK activity specifically in live cells in real time, we have designed a novel single-color FRET sensor for Rho-kinase (Figure 2A,B). The conformational change in the RhoKAR sensor associated with the activation of ROCK and phosphorylation of the substrate leads to a decrease in anisotropy, corresponding to an increase in FRET. Because of the higher sensitivity of anisotropy-based measurements and the large spectral occupancy of two-color FRET sensors, the single-color FRET approach is well-suited for measuring changes in dynamic cellular signaling systems such as the RhoA/ROCK pathway [71,72]. These anisotropy-based FRET biosensors enable us to sensitively measure the activity of multiple proteins simultaneously [73,74]. Anisotropy-based homotransfer FRET sensors enjoy superior performance compared to intensity-based heterotransfer FRET sensors by eliminating bleedthrough between donor and acceptor collection and improving measurement precision. Differing brightness in a two-color donor-acceptor FRET pair is of particular concern when measuring two-color FRET effectively. Emission from the dimmer fluorophore is particularly sensitive to noise, while emission from the brighter fluorophore can cause detector saturation [61]. Three-cube FRET, a common two-color FRET technique, requires multiple correction factors to be collected overall for appropriate quantification [61,166]. Reduced dynamic range has also been observed in two-color FRET pairs developed for biosensor multiplexing [167]. Anisotropy-based FRET reporters in live cells typically have a dynamic range of ~15 based on depolarized anisotropy measured after energy transfer and the measurement accuracy of 0.01 in a microscope [71]. We have previously reported that mVenus-based polarization-based FRET sensors show a higher dynamic range than those based on fluorescent proteins of other colors due to mVenus’s high extinction coefficient and quantum yield [72]. Because RhoKAR contains mVenus fluorophores, it benefits from these properties.

The RhoKAR sensor responded appropriately to RhoA activation with LPA (Figure 3A–C) and ROCK inhibition with Y27632 (Figure 3D–F). Because a previous ROCK-FRET sensor showed cross-reactivity to PKA [80], we validated that RhoKAR activation is insensitive to PKA activity with FSK stimulation. FSK treatment did not cause significant changes to RhoKAR activity (Figure 4A–C). Similarly, pretreatment of cells with PKA inhibitor H89 did not impact RhoKAR’s response to LPA stimulation (Figure 4D–F). A non-phosphorylatable mutant of the RhoKAR sensor, RhoKAR-DN, with the phosphorylation site on the ROCK substrate mutated from threonine to alanine. RhoKAR-DN showed a significantly lower response to LPA stimulation compared to the wild-type RhoKAR sensor (Figure 4G–I).

A previously developed ROCK sensor showed cross-reactivity to PKA. The ROCK phosphorylation sequence for this sensor was developed as a consensus sequence from the ROCK phosphorylation targets at Threonine 696 on protein phosphatase 1, Serine 1150 on ARHGAP35, and Threonine 567 on Ezrin [80]. Some of these sites are known to be phosphorylated by other kinases in addition to ROCK, with Ezrin Threonine 567 also targeted by PKA, PKC, LOK, and SLK [168,169]. The use of Adducin Threonine 445, which is only targeted by ROCK [83,84,85], as the substrate in RhoKAR (Figure 2C) offers improved specificity for ROCK activity.

Our results indicate that our sensor gives readout specific to Rho-kinase and that it is insensitive to activation or de-activation of PKA. Furthermore, our results suggest that the changes in anisotropy reported by the RhoKAR sensor are due to changes in sensor conformation in response to phosphorylation of the substrate by active ROCK. Because of the interest in ROCK as a drug target for diseases that represent a major public health burden, such as Alzheimer’s, stroke, metastatic cancer, and diabetic retinopathy [31,32,36,41,42,43,45,170], this sensor represents an important new tool for evaluating ROCK activity in live cells.

Future iterations of the RhoKAR sensor could be generated with fluorophores other than mVenus, such as a red fluorescent protein, to improve spectral compatibility with calcium reporter dyes such as Fluo-4 or Fura-2. While this version of the sensor can diffuse freely throughout the cell, future versions could incorporate localization tags to allow the study of ROCK activity restricted to specific subcellular compartments.

### 4.2. RhoKAR Reveals Compartmentalized Activation of ROCK During Cellular Protrusion and Retraction

Fluorescence-based biosensors allow for quantification of protein activity with high spatiotemporal resolution [171,172,173,174,175]. RhoA must be precisely spatiotemporally regulated for a variety of cellular processes to proceed effectively. During migration, RhoA is active at both the leading edge and the retracting tail edge [8,176,177,178]. RhoA is active at the furrow in the equatorial zone but inhibited in the polar regions during cytokinesis [102,103]. Additionally, RhoA helps strengthen cell-cell interaction during adhesion after initial cell-cell contact is established [103]. Despite the abundance of data detailing the fine spatiotemporal regulation of RhoA activity, whether RhoA’s effector ROCK shares these activation patterns is not as well understood.

In cells expressing the RhoKAR sensor, time course images at 40X overlaid with a FRET map reveal localized areas of high ROCK activity during cellular retraction and protrusion (Figure 5A,B, Supplemental Video S1). These areas of high RhoKAR activity illustrate the role of ROCK in cellular protrusion and retraction. RhoA’s role in driving changes to cell shape, mainly by modulating the activity and stability of the cytoskeleton, has been of interest for decades [179,180,181]. Because of RhoA/ROCK’s well-characterized role in promoting the active state of the actomyosin contractile apparatus via inhibitory phosphorylation of myosin light chain phosphatase [121,124,182,183,184,185], this localized increase in ROCK activity may be a significant driving force in the cytoskeletal processes pushing forward or pulling back the membrane.

### 4.3. Calcium Activates RhoA and ROCK in Fibroblasts via Calmodulin/CaMKII Signaling

Calcium is a vital second messenger in the cell [105,106,107,108,186]. Resting Ca^2+^ concentrations in the cytoplasm are in the nanomolar range, while the ER and extracellular space maintain Ca^2+^ concentrations in the millimolar range. Rapid influxes of Ca^2+^ to the cytoplasm or microdomains within the cell is used to drive fast processes and responses to signals such as GPCR engagement or cell depolarization [186,187,188]. Like RhoA activity, calcium must be precisely spatiotemporally regulated for physiological processes, such as proliferation and migration, to proceed effectively [95,146,189]. Recent findings in the literature have shown Ca^2+^-dependent activation of RhoA in a variety of tissue types, including vascular, sphincteric, and airway smooth muscle, as well as hippocampal neurons, cerebellar granule cells, embryonic kidney cells, endothelial cells, and intestinal epithelial cells [123,124,125,126,127,128]. Calcium also activates RhoA in disease states, including brain, colon, and endometrial cancer [129,130,131].

Our data indicate a tight relationship between RhoA/ROCK activity and intracellular calcium levels. RhoA/ROCK respond to increases and decreases in calcium in fibroblasts following ionomycin or EGTA treatment, respectively (Figure 6A–I). Notably, we also observed a steady increase in ROCK activity after ionomycin stimulation (Figure 6D), while RhoA response to ionomycin showed a biphasic pattern (Figure 6A). Our observations here may be a result of residual RhoA activity above baseline during the dip between activation phases or of multiple downstream consequences of ionomycin stimulation. Other groups have detailed signaling mechanisms where one initial signal activates multiple downstream mechanisms that converge RhoA activation, and such a mechanism could be at play here [134,190].

Ionomycin also activates RhoA in rabbit aortic vascular smooth muscle [145,191]. In this context, activation of RhoA by ionomycin was sensitive to treatment with W-7, a calmodulin inhibitor, but not KN93, a CaMKII inhibitor [145]. However, another group has reported that in the dendritic spines of rat hippocampal neurons, CaMKII inhibition was sufficient to prevent RhoA activation following glutaminergic signaling-dependent calcium influx [126]. We observed that ROCK activation by ionomycin in fibroblasts was sensitive to CaM inhibition with W-7 pretreatment (Figure 7A–C) and CaMKII inhibition with KN-93 (Figure 7D–F). The KN-93 sensitivity observed in mouse fibroblasts and rat hippocampal neurons, but not rabbit vascular smooth muscle, suggests that mechanisms for calcium-dependent activation are tissue-specific.

Taken together, our findings show that an ionomycin-dependent increase in intracellular calcium activates RhoA and ROCK via CaM and CaMKII. Although characterizing any GEFs potentially involved in the proposed signaling pathway was beyond the scope of this work, several GEFs are activated downstream of CaM and CaMKII, including ARHGEF2, ARHGEF23, ARHGEF24, and ARHGEF39 [192,193,194]. ARHGAP21 is a CaMKII target, and ARHGAP32 has been shown to be inactivated by CaMKII [194,195]. Other groups have observed transient, localized increases and decreases in RhoA activity due to changes in intracellular calcium during epithelial tight junction remodeling [196]. Our findings in fibroblasts have implications for processes such as cell migration, where an influx of calcium could promote RhoA/ROCK activity and the creation of contractile forces via cytoskeletal activation or reorganization.

## 5. Conclusions

In this work, we present the RhoKAR sensor, a new single-color FRET sensor that provides a readout of ROCK activity in real-time in living cells. Our work with this sensor in fibroblasts shows that RhoA/ROCK are activated by increasing intracellular calcium. ROCK activity downstream of calcium stimulation was sensitive to CaM and CaMKII inhibition, suggesting that these proteins are part of the signaling cascade that results in RhoA/ROCK activation after calcium stimulation in fibroblasts. The relationship between calcium-mediated ROCK activation and cell motility, both in migrating single cells and those collectively migrating, are important areas for future study.

## Data Availability

All data discussed are presented within the manuscript and Supplementary Materials. Data will be shared upon request by contacting Allison Mancini (allison.mancini@som.umaryland.edu). Biosensors developed for this paper will be made publicly available via Addgene.

## Supplementary Materials

The following supporting information can be downloaded at: https://www.mdpi.com/article/10.3390/s24216869/s1, Figure S1: RhoA-mCer3 biosensor schematic and sequence; Video S1: ROCK activity during 1-h imaging time course.
