# Supplementary material for: A Novel Single-Color FRET Sensor for Rho-Kinase Reveals Calcium-Dependent Activation of RhoA and ROCK
Source: Sensors (Basel). 2024 Oct 26;24(21):6869. doi: 10.3390/s24216869 (PMC11548655; doi:10.3390/s24216869)
Supplement: Supplementary file 1 [file sensors-24-06869-s001.zip › sensors-3189046_supplemental_figures.pdf]

A Novel Single-Color FRET Sensor for Rho-Kinase Reveals Calcium-Dependent Activation of RhoA and ROCK

Allison E. Mancini, Megan A. Rizzo

Material included: Supplemental Figure S1 with legend.

Supplemental Figure S1: RhoA-mCer3 biosensor schematic and sequence

A.

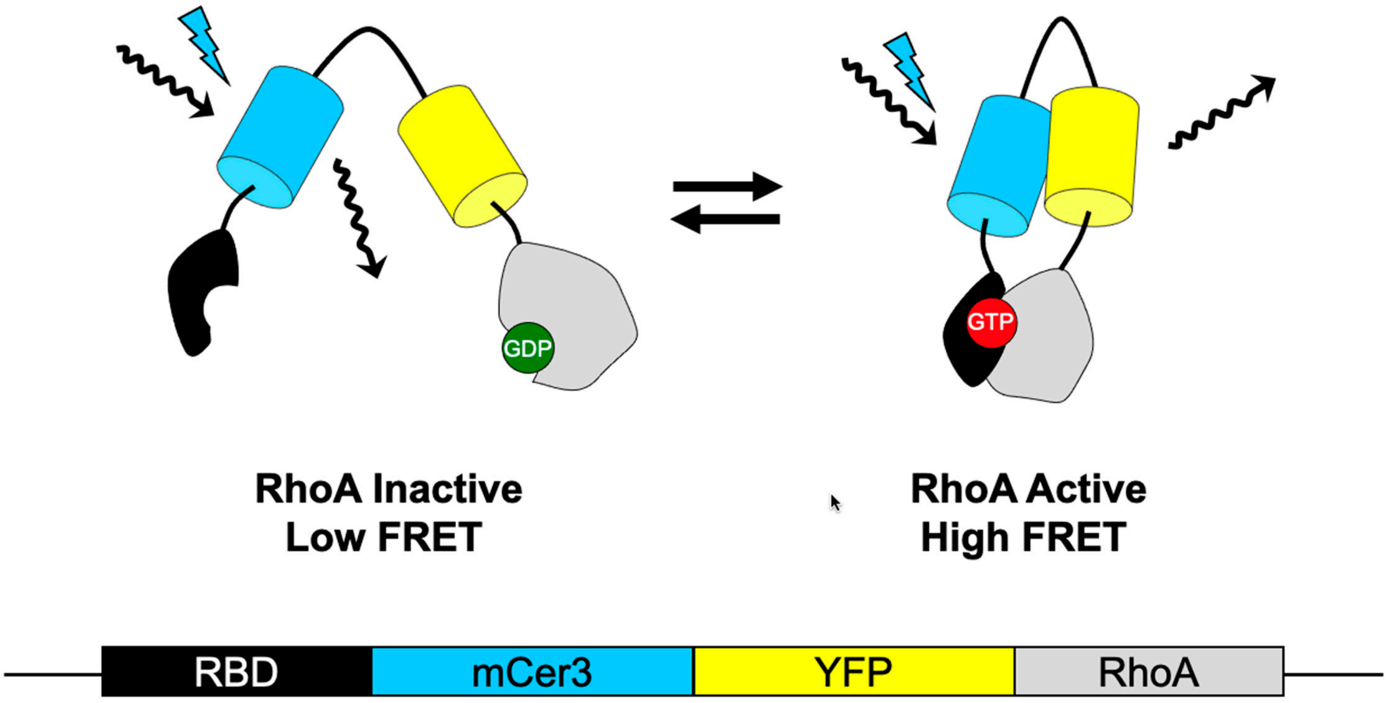

B. ATGGCACACCATCACCACCATCACGGTAGTGGCATCCTGGAGGACCTCAATATGCTCTACA
TCCGGCAGATGGCACTCAGCCTGGAGGACACAGAGCTGCAGAGGAAACTAGATCATGAG
ATCCGGATGAGGGATGGGGCCTGCAAGCTGCTGGCAGCCTGCTCCCAGCGAGAGCAGG
CTCTGGAAGCCACCAAGAGCCTGCTGGTGTGCAACAGCCGTATTCTCAGCTACATGGGTG
AGCTGCAGCGGCGAAAGGAGGCCCAGGTGCTGGAGAAGACAGGCGGGGGAATGGTGAG
CAAGGGCGAGGAGCTGTTCACCGGGGTGGTGCCCATCCTGGTCGAGCTGGACGGCGAC
GTAAACGGCCACAAGTTCAGCGTGTCCGGCGAGGGCGAGGGCGATGCCACCTACGGCA
AGCTGACCCTGAAGTTCATCTGCACCACCGGCAAGCTGCCCGTGCCCTGGCCCACCCTC
GTGACCACCCTGAGCTGGGGCGTGCAGTGCTTCGCCCGCTACCCCGACCACATGAAGCA
GCACGACTTCTTCAAGTCCGCCATGCCCGAAGGCTACGTCCAGGAGCGCACCATCTTCTT
CAAGGACGACGGCAACTACAAGACCCGCGCCGAGGTGAAGTTCGAGGGCGACACCCTG
GTGAACCGCATCGAGCTGAAGGGCATCGACTTCAAGGAGGACGGCAACATCCTGGGGCA
CAAGCTGGAGTACAACGCCATCCACGGCAACGTCTATATCACCGCCGACAAGCAGAAGAA
CGGCATCAAGGCCAACTTCGGCCTCAACTGCAACATCGAGGACGGCAGCGTGCAGCTCG
CCGACCACTACCAGCAGAACACCCCCATCGGCGACGGCCCCGTGCTGCTGCCCGACAAC

CACTACCTGAGCACCCAGTCCAAGCTGAGCAAAGACCCCAACGAGAAGCGCGATCACAT
GGTCCTGCTGGAGTTCGTGACCGCCGCCGGGATCACTCTCGGCATGGACGAGCTGTACA
AGGGATCTACTTCTGGTTCTGGTAAACCTGGTTCTGGTGAAGGTTCTACTAAAGGTGGTAG
TGGCGCGGCCGCTATGGTGAGCAAGGGCGAGGAGCTGTTCACCGGGGTGGTGCCCATC
CTGGTCGAGCTGGACGGCGACGTAAACGGCCACAAGTTCAGCGTGTCCGGCGAGGGCG
AGGGCGATGCCACCTACGGCAAGCTGACCCTGAAGTTCATCTGCACCACCGGCAAGCTG
CCCGTGCCCTGGCCCACCCTCGTGACCACCTTCGGCTACGGCCTGATGTGCTTCGCCCG
CTACCCCGACCACATGAAGCAGCACGACTTCTTCAAGTCCGCCATGCCCGAAGGCTACGT
CCAGGAGCGCACCATCTTCTTCAAGGACGACGGCAACTACAAGACCCGCGCCGAGGTGA
AGTTCGAGGGCGACACCCTGGTGAACCGCATCGAGCTGAAGGGCATCGACTTCAAGGAG
GACGGCAACATCCTGGGGCACAAGCTGGAGTACAACTACAACAGCCACAACGTCTATATC
ATGGCCGACAAGCAGAAGAACGGCATCAAGGTGAACTTCAAGATCCGCCACAACATCGAG
GACGGCAGCGTGCAGCTCGCCGACCACTACCAGCAGAACACCCCCATCGGCGACGGCC
CCGTGCTGCTGCCCGACAACCACTACCTGAGCTACCAGTCCGCCCTGAGCAAAGACCCC
AACGAGAAGCGCGATCACATGGTCCTGCTGGAGTTCGTGACCGCCGCCGGGATCACTCT
CGGCATGGACGAGCTGTACAAGGGGGGAATGGCTGCCATCCGGAAGAAACTGGTGATTG
TTGGTGATGGAGCCTGTGGAAAGACATGCTTGCTCATAGTCTTCAGCAAGGACCAGTTCC
CAGAGGTGTATGTGCCCACAGTGTTTGAGAACTATGTGGCAGATATCGAGGTGGATGGAA
AGCAGGTAGAGTTGGCTTTGTGGGACACAGCTGGGCAGGAAGATTATGATCGCCTGAGG
CCCCTCTCCTACCCAGATACCGATGTTATACTGATGTGTTTTTCCATCGACAGCCCTGATAG
TTTAGAAAACATCCCAGAAAAGTGGACCCCAGAAGTCAAGCATTTCTGTCCCAACGTGCC
CATCATCCTGGTTGGGAATAAGAAGGATCTTCGGAATGATGAGCACACAAGGCGGGAGCT
AGCCAAGATGAAGCAGGAGCCGGTGAAACCTGAAGAAGGCAGAGATATGGCAAACAGGA
TTGGCGCTTTTGGGTACATGGAGTGTTCAGCAAAGACCAAAGATGGAGTGAGAGAGGTTT
TTGAAATGGCTACGAGAGCTGCTCTGCAAGCTAGACGTGGGAAGAAAAAATCTGGTTGCC
TTGTCTTGTGA

C. MAHHHHHHGSGILEDLNMLYIRQMALSLEDTELQRKLDHEIRMRDGACKLLAACSQREQALE
ATKSLLVCNSRILSYMGELQRRKEAQVLEKTGGGMVSKGEELFTGVVPILVELDGDVNGHKFS
VSGEGEGDATYGKLTLKFICTTGKLPVPWPTLVTTLSWGVQCFARYPDHMKQHDFFKSAMPE
GYVQERTIFFKDDGNYKTRAEVKFEGDTLVNRIELKGIDFKEDGNILGHKLEYNAIHGNVYITAD
KQKNGIKANFGLNCNIEDGSVQLADHYQQNTPIGDGPVLLPDNHYLSTQSKLSKDPNEKRDH
MVLLEFVTAAGITLGMDELYKGSTSGSGKPGSGEGSTKGGSGAAAMVSKGEELFTGVVPILVE
LDGDVNGHKFSVSGEGEGDATYGKLTLKFICTTGKLPVPWPTLVTTFGYGLMCFARYPDHMK
QHDFFKSAMPEGYVQERTIFFKDDGNYKTRAEVKFEGDTLVNRIELKGIDFKEDGNILGHKLEY
NYNSHNVYIMADKQKNGIKVNFKIRHNIEDGSVQLADHYQQNTPIGDGPVLLPDNHYLSYQSA
LSKDPNEKRDHMVLLEFVTAAGITLGMDELYKGGMAAIRKKLVIVGDGACGKTCLLIVFSKDQF
PEVYVPTVFENYVADIEVDGKQVELALWDTAGQEDYDRLRPLSYPDTDVILMCFSIDSPDSLE
NIPEKWTPEVKHFCPNVPIILVGNKKDLRNDEHTRRELAKMKQEPVKPEEGRDMANRIGAFGY
MECSAKTKDGVREVFEMATRAALQARRGKKKSGCLVL*

Figure S1: RhoA-mCer3 biosensor schematic and sequence. **A**: The RhoA-mCer3 sensor consists of the Rho-binding domain (RBD) from Rhotekin, mCerulean3, YFP, and RhoA. When RhoA is inactive and in the GDP-bound conformation, the RBD cannot bind RhoA, and the two fluorophores are further apart, leading to low FRET between CFP and YFP. Upon activation of RhoA via exchange of GDP for GTP by a GAP, the RBD binds RhoA, bringing the two fluorophores closer together and causing an increase in C-Y FRET. **B**: RhoA-mCer3 FRET biosensor DNA sequence. **C**: RhoA-mCer3 FRET biosensor amino acid sequence.
